# Chloroplast vesicle transport

**DOI:** 10.1007/s11120-018-0566-0

**Published:** 2018-08-16

**Authors:** Emelie Lindquist, Henrik Aronsson

**Affiliations:** Department of Biological and Environmental Sciences, University of Gothenburg, Box 461, 405 30 Gothenburg, Sweden

**Keywords:** Chloroplast, Lipid, Membrane, Targeting, Transport, Vesicles

## Abstract

Photosynthesis is a well-known process that has been intensively investigated, but less is known about the biogenesis of the thylakoid membrane that harbors the photosynthetic machinery. Thylakoid membranes are constituted by several components, the major ones being proteins and lipids. However, neither of these two are produced in the thylakoid membranes themselves but are targeted there by different mechanisms. The interior of the chloroplast, the stroma, is an aqueous compartment that prevents spontaneous transport of single lipids and/or membrane proteins due to their hydrophobicities. Thylakoid targeted proteins are encoded either in the nucleus or plastid, and thus some cross the envelope membrane before entering one of the identified thylakoid targeting pathways. However, the pathway for all thylakoid proteins is not known. Lipids are produced at the envelope membrane and have been proposed to reach the thylakoid membrane by different means: invaginations of the envelope membrane, direct contact sites between these membranes, or through vesicles. Vesicles have been observed in chloroplasts but not much is yet known about the mechanism or regulation of their formation. The question of whether proteins can also make use of vesicles as one mechanism of transport remains to be answered. Here we discuss the presence of vesicles in chloroplasts and their potential role in transporting lipids and proteins. We additionally discuss what is known about the proteins involved in the vesicle transport and the gaps in knowledge that remain to be filled.

## Introduction

### Proplastids and chloroplasts

Proplastids can differentiate into a multitude of plastids, depending on conditions and the tissue in which they are present. In light exposed meristematic cells, proplastids differentiate to the well-known plastids: chloroplasts (Hurlock et al. 2014; Solymosi and Keresztes 2012; Vothknecht and Westhoff 2001). During differentiation, the poorly developed internal membrane system of proplastids with its many vesicles develops into thylakoid membranes with grana and stroma lamellae (Pribil et al. 2014; Solymosi and Keresztes 2012; Vothknecht and Westhoff 2001). In the absence of light, proplastids instead differentiate into etioplasts, with a characteristic membrane network (prolamellar body and prothylakoids). Upon illumination, etioplasts have the ability transform into chloroplasts, as the prolamellar body and prothylakoids are substituted for thylakoid membranes.

Plastids divide by binary fission mediated by specific proteins. As plastids divide four contractile rings surrounding the chloroplast are formed and after contraction two daughter plastids are formed (Osteryoung and Pyke 2014; Pyke 2007; Yoshida et al. 2012). Thylakoids extend through the contractile zone during early phases of chloroplast division, but separate from the zone in an unknown process before the two daughter plastids are formed (Osteryoung and Pyke 2014). That thylakoid membranes can be found in both daughter plastids after division (Osteryoung and Pyke 2014; Yoshida et al. 2012) is likely important as membranes in general almost exclusively are formed by growth and division, or fusion of already existing membranes (Cavalier-Smith 2000). A typical plant cell ranges between 20 and 100 µm in size, and chloroplasts are generally considered to measure ~ 5–10 µm (Solymosi and Keresztes 2012; Sundqvist and Ryberg 1993; Taiz and Zeiger 2010).

The lipid composition of chloroplast membranes differs from other membranes of the cell. Chloroplasts mostly contain glycolipids and sulpholipids, in contrast to extraplastidial membranes which main components are phospholipids (Andersson and Dörmann 2009; van Meer et al. 2008). The composition of chloroplast membranes is much similar to the thylakoid membranes of cyanobacteria, reflecting its endosymbiotic origin (Cavalier-Smith 2000).

### Thylakoid biogenesis and maintenance

The chloroplast envelope membranes, surrounding the chloroplast, contain mainly galactolipids, ≥ 60 mol%, but also phospholipids and sulpholipids. Of the two existing galactolipid forms, the rod-shaped digalactosyl diacylglycerol (DGDG) is more prominent in the outer envelope, whereas the cone-shaped monogalactosyl diacylglycerol (MGDG) is more prominent in the inner envelope, which contribute to the shape and function of the membranes (Andersson and Dörmann 2009).

Both DGDG and MGDG are assembled in the envelope membrane (Andersson and Dörmann 2009; Benning 2009; Kobayashi 2016) and most DGDG is produced in the outer envelope where also the DGDG synthases (DGD1 and DGD2) can be found (Dörmann and Benning 2002; Kelly et al. 2016). Of the three MGDG synthases, MGD2 and MGD3 are located in the outer envelope whereas MGD1 is found in the inner envelope and is also the main producing synthase.

The lipid composition of the outer envelope membrane is more similar to the extraplastidial membranes and possesses a greater negative charge than the inner envelope membrane, which is producing lipids for the plastid inner membranes, e.g., the thylakoids. The similarity of composition of the inner envelope membrane and thylakoids is therefore logical (Andersson and Dörmann 2009). Proteins are also part of the membranes with acyl lipids to protein ratio of 2.3–3:1 in the outer envelope and 0.8–1:1 at the inner envelope. The thylakoids are more protein dense with a ratio of 0.4:1 (Block et al. 1983).

Thylakoid membranes are inherited from one generation to another, but are likely maintained by invagination of the inner envelope membrane and/or by vesicles providing a supply of lipid (Lindquist et al. 2016). In addition to lipids, thylakoids also contain proteins and pigments. The membranes can vary in structure depending on the developmental stage of the plant, e.g., there are differences between young and old plastids. Chlorophylls are the major pigments in chloroplasts. Although carotenoids are also present, these are most often masked by the heavy abundance of chlorophylls.

DGDG and MGDG are the main components found in the thylakoid bilayer membranes, where DGDG and MGDG are enriched in the inner and outer leaflet, respectively (Rawyler et al. 1987). These lipids are important as structural components for the photosystems (PSI and PSII) to maintain a functional photosynthetic process (Kobayashi 2016). DGDG has been shown to have a role in function, stability and structure of PSI and PSII, whereas mutants with highly reduced MGDG levels (~ 80%) show strongly impaired PSII activity. Moreover, MGDG can facilitate photoprotection and oligomerization of light harvesting complex II (LHCII), as well as dimerization of PSII (Kobayashi 2016).

The thylakoid membranes are continuous, and the presence of integral carotenoids and transmembrane spanning proteins results in a stable bilayer (Andersson and Dörmann 2009). The membranes surround a densely packed luminal space (Kieselbach et al. 1998; Pribil et al. 2014; Shimoni et al. 2005; Weibull and Alertsson 1988) that mostly contains the oxygen evolving complex (Kirchhoff et al. 2011).

### Lipid transport

Different non-exclusive models are proposed regarding lipid transfer from the inner envelope membrane through the stroma to the thylakoid membrane. One considers soluble lipid transfer proteins, a second proposes direct contact between the inner envelope membrane and the thylakoid membrane that can occur via invaginations of the inner envelope, and a third relates to vesicle transport (Hurlock et al. 2014; Pribil et al. 2014; Rast et al. 2015). Here we discuss the support for these models, with major focus on vesicle transport.

### Lipid transfer proteins

Lipid transfer proteins are small in size (~ 9 kDa) and considered mainly to mediate transfer of cuticular lipids. Many lipid transfer proteins are localized to the cell walls, plasma membranes, and surface waxes (Hurlock et al. 2014). In chloroplasts, one observation has been reported of lipid transfer proteins in rough lemon (*Citrus jambhiri* Lush), but their role was not thought to be the transfer of bulk of lipids for thylakoid maintenance or biogenesis. Rather they were speculated to be important for chloroplast protection and repair, as well as biosynthesis and transfer of lipids over short distances (Nishimura et al. 2008). Thus, there is no clear evidence for a lipid transfer protein mechanism in chloroplasts, a point of view also reflected in recent reviews of the subject (Pribil et al. 2014; Rast et al. 2015).

### Direct contact of membranes

Invaginations of the inner envelope protruding into the stroma were observed early on using transmission electron microscopy (TEM) (Mühlethaler and Frey-Wyssling 1959). Direct contact between the envelope membrane and the thylakoid membrane has been observed in lettuce (*Lactuca sativa*), where the stroma lamellae was attached to the inner envelope (Shimoni et al. 2005). However, invaginations of the inner envelope membrane have been more often observed than direct contact sites when using TEM. Invaginations have been proposed to occur only in young undifferentiated proplastids and chloroplasts, where it would be the main source of lipid transfer during thylakoid assembly (Andersson and Dörmann 2009; Vothknecht and Westhoff 2001). Lack of invagination observations in mature plant and cyanobacteria chloroplasts has been interpreted to mean that invaginations do not occur at all in older tissue (Hurlock et al. 2014; Vothknecht and Westhoff 2001). However, some rare observations of invaginations have been made in mature chloroplast, e.g., in pea (Morré et al. 1991). Due to its low frequency, it can be assumed that invaginations occur more often in young than mature plastids. When invaginations in pea were observed, vesicles were noted as well, suggesting that the two different mechanisms can co-exist and are non-exclusive (Hurlock et al. 2014; Lindquist et al. 2016; Morré et al. 1991). So far, no proteins have been identified to regulate or mediate the invagination mechanism (Hurlock et al. 2014).

### Vesicle transport

**Fig. 1:**
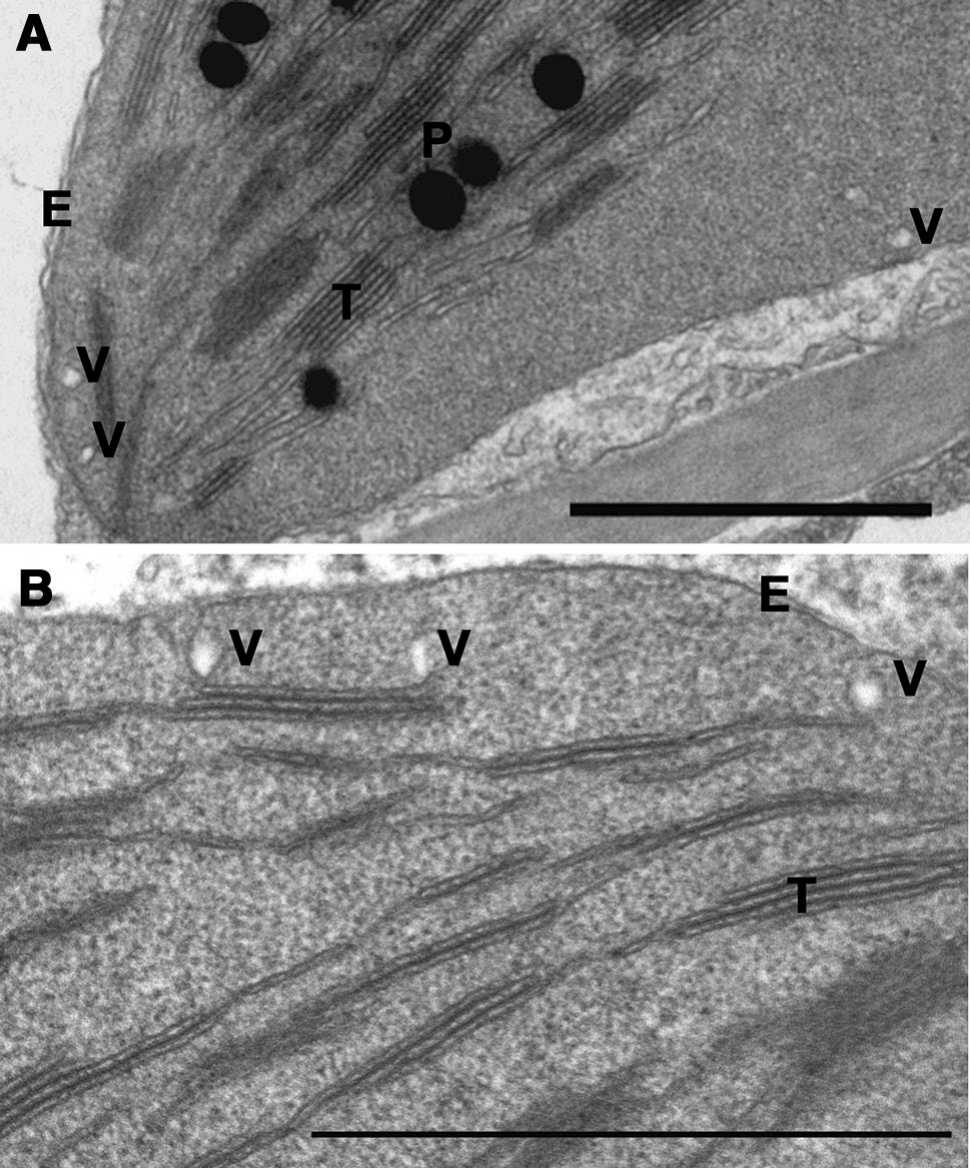
Vesicle appearance in chloroplasts. Chloroplasts from **A** lettuce (*Lactuca sativa*) and **B** Arabidopsis (*Arabidopsis thaliana*) leaves with vesicles present. E, envelope membrane; T, thylakoid membrane; P, plastoglobules; V, vesicle. Scale bar: 1 µm. Micrograph courtesy to Katalin Solymosi, Eötvös Loránd University, Hungary

Vesicles have been observed by TEM in chloroplasts (Fig. 1) (Garcia et al. 2010; Karim et al. 2014; Morré et al. 1991; Westphal et al. 2001b, 2003) and in other plastid types (Lindquist et al 2016). The formation of vesicles in general (e.g., COPI, COPII and clathrin pathway) is mediated by protein interactors (Bassham et al. 2008). Subsequently, several proteins have been proposed to mediate vesicle transport from the inner envelope membrane to thylakoids (Andersson and Sandelius 2004; Garcia et al. 2010; Karim et al. 2014; Khan et al. 2013; Kroll et al. 2001; Tanz et al. 2012). Accordingly, vesicles are considered to maintain thylakoid membranes in mature plastids (Andersson and Dörmann 2009; Rast et al. 2015; Vothknecht and Westhoff 2001). In developing plastids, vesicles have also been discussed as a lipid transfer mechanism (Andersson et al. 2001; Räntfors et al. 2000; Tanz et al. 2012; Wang et al. 2004) and have been observed in proplastids (Lindquist et al. 2016; Solymosi and Keresztes 2012; Vothknecht and Westhoff 2001). Since both vesicles and invaginations have been observed in young and mature chloroplasts, at least two lipid transfer mechanisms can be postulated to occur at different developmental stages (Andersson et al. 2001; Hurlock et al. 2014; Lindquist et al. 2016).

Support for models of lipids being transported with vesicles comes from impairment of galactolipid movement from envelope to thylakoids at low temperature, which is a well-known phenomenon from cytosolic vesicle transport. In low temperature treated chloroplasts, vesicles do accumulate in the stroma, similar to observations of vesicles in the cytosolic system (Andersson et al. 2001; Morré et al. 1991). However, vesicles do occur in chloroplast of plants grown at ambient temperatures. This argues against the idea that vesicles are artifacts of low temperature treatment, but instead supports vesicles as persistent features that are present regardless of temperature (Lindquist et al. 2016). Galactolipid movement from the inner envelope requires stromal protein(s), and is stimulated by ATP and GTP, similar to cytosolic vesicle transport (Andersson et al. 2001; Räntfors et al. 2000). Interestingly, within stromules, a directional ATP-dependent transport with batches of GFP was observed to move at 0.12 µm/s (Kohler et al. 2000), and the batches were proposed to be vesicles. Assuming movement of vesicles from the envelope to the thylakoid at the same speed, this distance would be covered within a second(s). Thus, one could speculate that the low frequency of vesicles observed in chloroplast using TEM is because their movement is rather fast and lifetime is relatively short. In addition, the need for of vesicles might vary with development and environmental conditions.

It has been discussed that spherical structures observed in chloroplasts using TEM might be either cross-sections of tubules or vesicles, since TEM often is two-dimensional (Lindquist et al. 2016). However, the use of dual-axis TEM and scanning-TEM tomography has clearly revealed spherical vesicles by 3D imaging (Charuvi et al. 2012). Tubules were also observed, but they have a different diameter than do vesicles (Charuvi et al. 2012); tubules being ~ 35–40 nm in diameter whereas vesicles were ~ 50 nm in diameter. Serial sectioning using TEM has also shown presence of vesicles (Westphal et al. 2001b). Thus, experimental evidence exists for vesicle structures in chloroplasts, and not all spherical structures can be attributed to cross-sectioned tubules.

Vesicle transport seems to be a eukaryotic phenomenon, as it has still not been clearly demonstrated in prokaryotic cyanobacteria (Liberton et al. 2006; Rast et al. 2015; Westphal et al. 2003; Vothknecht and Westhoff 2001). Interestingly, a bioinformatics study in *Synechocystis* identified a homolog of a vesicle-related protein in yeast, although experimental verification is still needed (Keller and Schneider 2013). A second preliminary bioinformatics study could not verify the major vesicle core components in cyanobacteria (unpublished observation Lindquist E, Aronsson H). Vesicle structures have been observed in *Microcoleus* sp., but with a diameter much larger than chloroplast vesicles, 150–300 nm compared to 30–70 nm, respectively (Nevo et al. 2012; Westphal et al. 2001b).

However, no such structures have been observed in other investigated cyanobacteria (Nevo et al. 2012; Westphal et al. 2003). In addition, neither vesicles nor invaginations were observed during thylakoid formation in 4–5 days old cultures of *Synechocystis* (Liberton et al. 2006). Thus, thylakoid formation with assistance of vesicles in cyanobacteria remains to be demonstrated.

### Cytosolic versus chloroplastic vesicle system

**Fig. 2:**
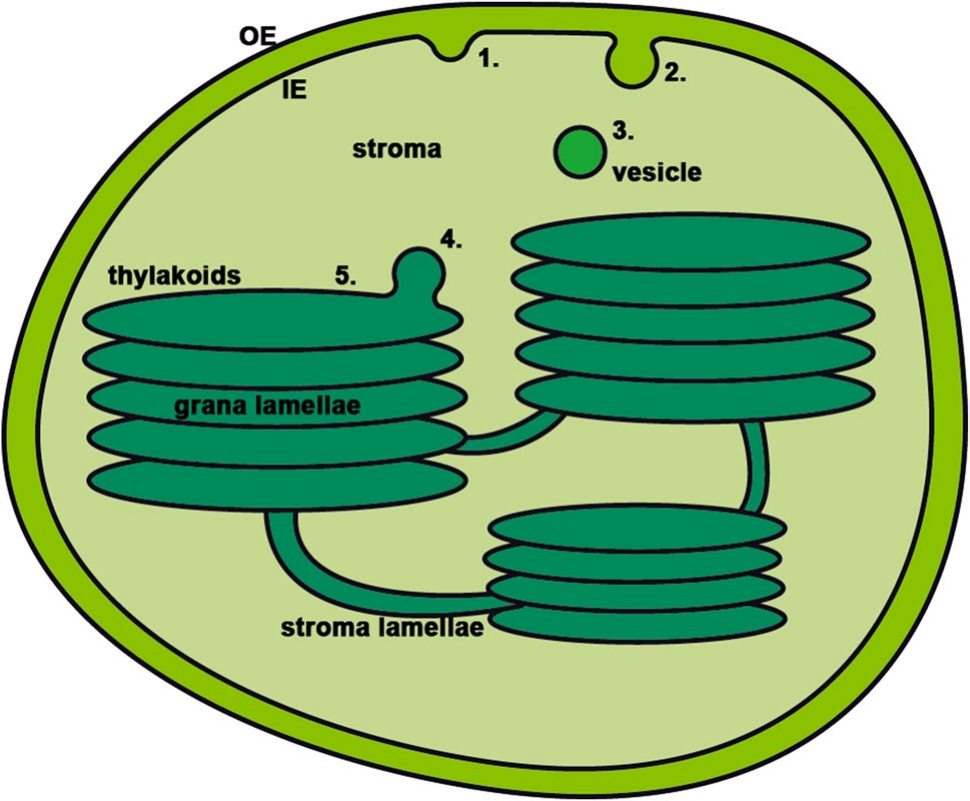
Vesicle transport in chloroplasts. The chloroplast is surrounded by the outer envelope (OE) and inner envelope (IE) membrane, where the latter act as donor membrane for vesicles. The thylakoid membranes consist of grana lamellae and stroma lamellae, and are the target membrane of vesicles. The vesicle process from initiation at the donor membrane to the fusing step at the target membrane is shown. The vesicle is initiated (1) and buds of (2) from the inner envelope where CPSAR1, FZL, THF1, and VIPP1 are found. The vesicle then moves through the stroma (3) where CPRabA5e, CPSAR1 and THF1 are located. Finally, the vesicle gets tethered (4) and fused to the target membrane (5) where CPRabA5e, CURT1, FZL, SCO2, THF1 and VIPP1 are placed

Intracellular vesicle transport of lipids and proteins from the donor to the target membrane is selective and fast (Kirchhausen 2000). This transport is composed of three different vesicle systems: coat protein complex I and II (COPI and COPII) vesicles, and clathrin coated vesicles (CCV). The COPI/II and CCV systems have been mainly characterized in yeast and mammals, but are generally considered to function in plants also (Bassham et al. 2008) (but see Robinson et al. 2015). All three coated cytosolic vesicle systems follow a general common procedure, but use different sets of proteins for execution (Bassham et al. 2008; Kirchhausen 2000). At the donor membrane vesicles are initiated, then coat proteins and possible cargo are added, and finally it buds off from the membrane. Before reaching the target membrane the coat is shed, and at the target membrane the vesicle is tethered and fuses with the membrane and its cargo is unloaded (Fig. 2).

The observed size (diameter) of vesicles in the cytosol is often between ~ 50–100 nm but exceptions occur. COPI vesicles are ~ 60–100 nm (Faini et al. 2013), COPII ~ 60–90 nm but as large as 500 nm has been observed (Brandizzi and Barlowe 2013; Gurkan et al. 2006). CCVs have been observed with flexible sizes up to 200 nm, although in plants and yeast they are generally smaller, 35–60 nm (McMahon and Boucrot 2011). In comparison, chloroplast vesicles are in the range of 30–70 nm (Garcia et al. 2010; Karim et al. 2014; Lindquist et al. 2016; Westphal et al. 2001b).

If the chloroplast vesicle system has similarities with cytosolic systems, proteins that maintain cytosolic vesicle transport could be expected to have homologs in chloroplasts, with similar functions and a degree of sequence conservation (Andersson and Sandelius 2004). Using bioinformatics with Arabidopsis as model plant revealed that most cytosolic COPII proteins had putative homologs in the chloroplast, whereas only a few potential COPI and CCV homologs were found (Khan et al. 2013; Lindquist et al. 2014). Moreover, analysis of motifs for cytosolic cargo proteins identified several putative cargo proteins destined for the thylakoids. The majority of these were linked to photosynthesis, e.g., light harvesting complex proteins (Khan et al. 2013), which had already been proposed to use vesicles for transport (Tanz et al. 2012). While an almost complete putative COPII system was identified by bioinformatics, only two proteins have been experimentally validated, CPSAR1 and CPRabA5e. Many of the predicted protein have already been shown to be located elsewhere in the cell, and thus may either be dual targeted or wrongly localized in earlier studies. This makes it less likely that the chloroplast vesicle system is a complete COPII system, but instead may be a unique system possibly integrating proteins similar to the COPI and CCV systems.

### Proteins involved in chloroplast vesicle transport

Several putative proteins have been suggested to be actors in chloroplast vesicle transport and in thylakoid biogenesis, as accumulation or deletion of vesicles have been observed in mutant plants lacking these proteins. Although the precise roles for these proteins are still to be resolved, we present below seven proteins which have all been linked to vesicle transport and/or thylakoid biogenesis (Table 1; Fig. 1).

**Table 1:** Chloroplast vesicle proteins found in Arabidopsis

Arabidopsis protein (accession no.)	Possible role based on chloroplast localization	Envelope membrane	Stroma	Thylakoid membrane	Mutant phenotype in Arabidopsis	References
CPRabA5e (At1g05810)	Interacts with the vesicle before the fusion step and onwards	No	Yes	Yes	No visible phenotype	(Karim et al. 2014)
CPSAR1 (At5g18570)	Forming vesicles, recycled	Yes (inner)	Yes (vesicle)	No	Embryo-lethal, non-viable	(Bang et al. 2009 Chigri et al. 2009; Garcia et al. 2010)
CURT1 (At4g01150, A) (At2g46820, B) (At1g52220, C) (At4g38100, D)	Fusion of vesicles	No	No	Yes	No visible phenotype	(Armbruster et al. 2013)
FZL (At1g03160)	Fission and fusion of vesicles	Yes (inner)	No	Yes	Pale green leaves, delayed development	(Gao et al. 2006)
SCO2 (At3g19220)	Fusion of vesicle	No	No	Yes	Pale green/albino cotyledon	(Muranaka et al. 2012; Shimada et al. 2007; Tanz et al. 2012)
THF1 (At2g20890)	Fission, and interacts with the vesicle before the fusion step and onwards	Yes	Yes	Yes	Variegated leaves, delayed development	(Huang et al. 2006; Wang et al. 2004)
VIPP1 (At2g20890)	Fission and fusion of vesicles	Yes (inner)	No	Yes	Pale green leaves, deficient photosynthesis	(Aseeva et al. 2007; Kroll et al. 2001; Otters et al. 2013; Zhang et al. 2012)

### CPRabA5e

The chloroplast (= CP) Ras-related in brain GTPase (Rab), CPRabA5e, is a GTPase with proven GTPase activity, and with a chloroplast transit peptide to enable targeting to stroma and thylakoid membranes (Table 1) (Karim et al. 2014). CPRabA5e has similarities with Rab proteins, and is therefore proposed to anchor to the membrane by a geranylgeranylation motif (Stenmark 2009). CPRabA5e is related to Rab11, which in animal cells is linked to recycling endosomes that receives material from the early endosomes and pass it on to the cell surface or to the trans-Golgi network. However, no recycling endosomes have been characterized in plant cells (Contento and Bassham 2012). Rab proteins have several roles as molecular switches and in regulation of effector proteins through GTP/GDP binding and hydrolysis, but their major role involves controlling vesicles related to membrane transport. Thus, Rab proteins are involved in most steps in vesicle transport, i.e., cargo sorting, vesicle budding, movement, uncoating, tethering, and fusion (Nielsen et al. 2008; Rutherford and Moore 2002; Stenmark 2009).

CPRabA5e was shown to complement yeast mutants deficient of Rab Ypt31/31, proteins known to regulate vesicle transport in exo- and endocytosis in yeast (Chen et al. 2011; Karim et al. 2014). Following preincubation at 4 °C, chloroplasts in *cprabA5e* Arabidopsis knockout plants showed more vesicles accumulated close to the envelopes, and an altered thylakoid morphology similar to THF1 mutants (Wang et al. 2004). Larger plastoglobules were also displayed, compared to wild type. Moreover, during oxidative stress more vesicles were observed in *cprabA5e* and growth was more impaired in *cprabA5e*, both compared to wild type (Karim et al. 2014).

A yeast two-hybrid screen using CPRabA5e as bait identified fourteen possible protein interactors involved in development, photosynthesis, and stress; these proteins were located in the thylakoid membranes, plastoglobules, and the stroma (Karim et al. 2014). Interestingly, among the proteins identified were LHCB1 and LHCB3 belonging to light harvesting complex. These have previously been suggested as vesicle cargo proteins (Khan et al. 2013; Tanz et al. 2012). However, attempts to validate interaction of LHCB3 and CPRabA5e using bimolecular fluorescence complementation (BiFC) have so far not been successful (unpublished observation, Lindquist E, Karim S, Aronsson H) and remains to be further investigated.

CPRabA5e shows expression throughout the plant life cycle but has its highest expression during seed germination and the seedling stages (Alezzawi et al. 2014; Karim et al. 2014). The expression pattern is similar to the one found for CPSAR1 (Garcia et al. 2010) and supports the presence of vesicles at all developmental stages.

### CPSAR1

The GTPase Secretion associated Ras1 (SAR1) protein regulates initial steps during vesicle budding in the cytosol of mammals, plants and yeast. A protein, CPSAR1, with amino acid sequence and protein domain similarities to SAR1 was identified in Arabidopsis and shown to be localized in chloroplasts (Andersson and Sandelius 2004; Garcia et al. 2010). CPSAR1 is a GTPase with demonstrated GTPase activity, is involved in thylakoid biogenesis, and has been observed in the chloroplast envelope, stroma, and in vesicles (Table 1; Fig. 2) (Bang et al. 2009; Garcia et al. 2010). When fused with GFP a punctuate pattern of CPSAR1 assumed to be due to dimerization was observed in chloroplasts (Bang et al. 2009). However, this punctate pattern could support localization of CPSAR1 to vesicles, as a similar pattern previously shown in stromules was proposed to be vesicles (Kohler et al. 2000).

Arabidopsis mutants lacking CPSAR1 show lethality as embryo maturation is arrested, while RNAi mutants with reduced levels of CPSAR1 have partially developed thylakoids (Chigri et al. 2009; Garcia et al. 2010). CPSAR1 has also been named AtOBGL and AtObgC (Bang et al. 2009; Chigri et al. 2009). CPSAR1 presumably originated from the bacterial Obg (SpoOB-associated GTP-binding protein) protein subfamily, and in accordance with the suggested function of this group, CPSAR1 has been assigned roles in protein synthesis and ribosome biogenesis within the chloroplast (Bang et al. 2012; Brandizzi 2011; Chigri et al. 2009). In addition, Obg proteins have also been suggested to play a role in sporulation processes, which involves membrane trafficking (Brandizzi 2011). The crystal structure of SAR1 shows a Sar1–NH2-terminal activation recruitment (STAR) motif, which enables interaction with the Guanine nucleotide Exchange Factor (GEF) Sec12. SAR1 also contains a coat protein interacting alpha helix, followed by GTPase domains in its N-terminus (Huang et al. 2001). Interestingly, the STAR motif contains nine bulky hydrophobic amino acids that can vary between species, but using PROSITE (prosite.expasy.org) one cannot find this motif in yeast and Arabidopsis SAR1. The STAR motif in SAR1 includes a three amino acid combination of phenylalanine (F), isoleucine (I), leucine (L), tryptophan (W), or valine (V). Yeast only shows a combination of two of the amino acids, isoleucine and leucine (IL) (Huang et al. 2001), whereas for Arabidopsis SARA1 and SARA1B, a putative three amino acid combination is identified: phenylalanine, leucine, and phenylalanine (FLF), and is located in the N-terminus. PROSITE predicts SARA1A and SARA1B to be part of the small GTPase Sar1 family, thus similar to SAR1 of yeast. However, CPSAR1 belongs to the GTP1/Obg family and has no IL or FLF prior to the coiled-coil domain, but instead contains an amino acid combination consisting of two leucines (LL). If the LL represents a true STAR motif and the coiled-coil domain of CPSAR1 provides the same function as the alpha helix in SAR1, then the differences between these proteins would be minimized; this remains to be demonstrated.

Alignment of CPSAR1 and SAR1 shows CPSAR1 to have an extended *N*-terminus with approximately 200 unique amino acids. This may represent an adaptation to new cellular functions, perhaps specifically in chloroplasts (Brandizzi 2011). Developmental arrest in mutants lacking CPSAR1 (Garcia et al. 2010) shows the importance of this protein, and its presence in vesicles cannot be solely explained by it having a ribosomal role.

### CURT1

The Arabidopsis curvature thylakoid 1 (CURT1) proteins (A-D) form oligomers and are located in the grana margins of the thylakoid membranes (Table 1; Fig. 2). CURT1 proteins have been shown to maintain membrane curvature, grana architecture, and formation (Armbruster et al. 2013). When analyzing different loss of function mutants (double, triple, quadruple) thylakoids are observed to be wider and more curved, with lack of wild-type grana structures. The double mutant *curt1ac* accumulated vesicles in the stroma and close to the envelope, and these vesicles can be speculated to be part of the thylakoid formation. Thus, CURT1 proteins are assumed to be important for functional vesicle transport in chloroplasts (Armbruster et al. 2013; De Alda et al. 2014).

### FZL

In Arabidopsis one FZL (FZO like) protein exists, which has coil-coiled, GTPase domains, and transmembrane helices similar to the fuzzy onion (FZO) protein (Gao et al. 2006). FZO belongs to the dynamin superfamily of remodeling GTPases, and is located in the outer mitochondria membrane. In animals and fungi it mediates fusion of apposing mitochondria outer membranes through its coiled-coil domains that are a typical feature of dynamin related proteins (Koshiba et al. 2004; Meeusen and Nunnari 2005). Dynamin is important for vesicle budding at the donor membrane in the clathrin coated pathway (Dannhauser and Ungewickell 2012). However, the Arabidopsis FZL has low homology with the mitofusion and dynamin (DYN1) domain found in the FZO family, which might explain why FZL affects chloroplast morphology and not mitochondria morphology (Gao et al. 2006). FZL is located at the envelope and the thylakoid membranes where it is anchored by two transmembrane domains at the *C*-terminal part of the protein (Table 1; Fig. 2). This leaves the coiled-coil and GTPase domains protruding into the stroma (Gao et al. 2006). It is not known if the coil-coiled could work as for FZO i.e., to fuse membranes. But interestingly, vesicles accumulate in *fzl* mutants and do not seem to fuse with the thylakoid membranes. It could therefore be speculated that this dynamin coil-coiled region has a function in fusing membranes, similar to FZO. Chloroplasts of *fzl* appear larger and with an unusually shape, abnormal proportions of stroma, and grana thylakoids, compared to wild-type chloroplasts. Also, overexpression of FZL in chloroplasts results in defects in the thylakoid organization. Although the exact function is still to be fully resolved, FZL appear to be a special protein within the dynamin superfamily of membrane-remodeling GTPases, assumed to facilitate membrane fusion processes and thereby maintain a dynamic but organized thylakoid network (Gao et al. 2006).

### SCO2

The Arabidopsis Snowy cotyledon 2 (SCO2) is a disulphide isomerase found in thylakoid membranes (Fig. 2) that facilitates integration of photosynthesis related proteins, e.g., LHCB1. Mutants lacking SCO2 initially display pale/green albino cotyledons with impaired chloroplast biogenesis, but they turn into green leaves during development (Table 1) (Albrecht et al. 2008; Muranaka et al. 2012; Shimada et al. 2007; Tanz et al. 2012). Chloroplasts observed in cotyledons of *sco2* mutants appear both globular and normal. Vesicles accumulate mainly close to the inner envelope at the rounded ends of elongated chloroplasts compared to wild-type chloroplasts (Tanz et al. 2012). Vesicle formation and movement during thylakoid biogenesis was impaired in absence of SCO2, resulting in vesicle accumulation in the mutants. Furthermore, LHCB1 was shown to interact with SCO2 and subsequently hypothesized to travel from envelope to thylakoid using vesicles (Tanz et al. 2012).

### THF1

The Arabidopsis Thylakoid formation 1 (THF1) protein is present in the stroma and the thylakoid (Table 1; Fig. 2). It was suggested to have role in vesicle fusion at the thylakoid membrane as mutants lacking THF1 showed a variegated leaf pattern with accumulation of vesicles and lack of thylakoid membranes in the white/yellow leaf patches of leaves (Wang et al. 2004). The green leaf sectors contained chloroplasts with inner structures both disturbed and normal. The impaired thylakoid organization was mainly visible in young seedlings, and the presence of a mix with normal structures suggests that the inhibitory effect of THF1 is compensated by an unknown mechanism (Wang et al. 2004).

THF1 has also been shown to interact with LHCB proteins (Huang et al. 2013), which is interesting considering that LHCBs have been proposed to be a possible cargo in vesicles (Khan et al. 2013; Tanz et al. 2012). THF1 has also been named Psb29 and proposed to play a role in not only PSII biogenesis, but also in pathogen defense and sugar signaling (Huang et al. 2006, 2013). As THF1 was further investigated it appeared in the outer envelope membrane and stroma but not in thylakoids, in contradiction to previous localization data (Table 1; Fig. 2) (Huang et al. 2013). However, the dual location could indicate different roles of THF1, when in the outer envelope membrane it is involved in sugar signaling and when in the stroma it is part of the vesicle transport system (Huang et al. 2006, 2013; Keren et al. 2005).

### VIPP1

The Vesicle inducing protein in plastids 1 (VIPP1) has been found in algae, cyanobacteria, and plants, all having oxygenic photosynthesis (Nordhues et al. 2012; Vothknecht et al. 2012; Vothknecht and Westhoff 2001). In Arabidopsis VIPP1 is located at the envelope and thylakoid membranes (Table 1; Fig. 2) (Kroll et al. 2001; Vothknecht et al. 2012). Initially, the suggested function of VIPP1 was to mediate lipid transport between envelope and thylakoid membranes, as supported by mutant analyses in Arabidopsis and cyanobacteria (Kroll et al. 2001; Westphal et al. 2001a; Vothknecht et al. 2012). Arabidopsis mutants with reduced levels of VIPP1 display fewer vesicles, impaired thylakoid biogenesis, and a disturbed photosynthetic electron transport chain compared to wild type (Kroll et al. 2001). Similarly, in cyanobacteria a reduced level of VIPP1 resulted in impaired thylakoid biogenesis and deficient photosynthesis (Westphal et al. 2001a; Vothknecht et al. 2012). Thus, VIPP1 was proposed to play a role in thylakoid biogenesis enabling vesicle formation. VIPP1 can be observed to assemble into ring structures and at high concentrations form rod-like structures resembling microtubules (Vothknecht et al. 2012).

VIPP1 is of prokaryotic origin and has a bacterial homolog, the phage shock protein A (PspA) in non-photosynthetic bacteria, which it has evolved via gene duplication (Westphal et al. 2001a; Vothknecht et al. 2012). The possible function of VIPP1 has been expanded as further investigations support a role more related to PspA functioning as a membrane stabilizer (Pribil et al. 2014; Vothknecht et al. 2012).

In relation to photosynthesis, Vipp1 deficiencies are linked to incomplete assembly of photosystem components, as from studies in cyanobacteria and single cell algae (Gao and Xu 2009; Nordhues et al. 2012), or perturbed thylakoid formation *per se*, as from studies in Arabidopsis and cyanobacteria (Aseeva et al. 2007; Kroll et al. 2001; Westphal et al. 2001a). VIPP1 has also been shown to interact with Alb3.2 and enhances substrate binding to receptors in the Tat pathway (Nordhues et al. 2012; Pribil et al. 2014). VIPP1 was first suggested to be membrane bound but is now suggested to appear also as a soluble protein similar to PspA (Nordhues et al. 2012). Although VIPP1 is obviously an important chloroplast protein, its exact function is not simple to resolve; rather it presents a complicated story in need of further investigation.

### Proteins as vesicle cargo targeted to the thylakoids

The majority of proteins residing in the chloroplast are nucleus-encoded (~ 95%), and translated in the cytosol before entering the chloroplast using the general protein import machinery (Aronsson and Jarvis 2008). However, some nucleus-encoded chloroplast proteins use another pathway to pass the envelope membranes. In algae, cytosolic proteins have been shown to use vesicles from the endomembrane system for targeting their chloroplast destination (Radhamony and Theg 2006), and in plants the carbonic anhydrase (CAH1) and nucleotide pyrophosphatase/phosphodiesterase 1 (NPP1) have been shown to use an unusual ER to chloroplast targeting pathway (Nanjo et al. 2006; Villarejo et al. 2005). CAH1 and NPP1 have been proposed to reach chloroplast envelope by vesicles, but no clear mechanism have been presented. One model proposes that vesicles fuse to the envelope membrane and proteins enter the chloroplast either by the TOC/TIC translocases, or by an unknown translocase or even vesicles formed at the inner envelope, (Radhamony and Theg 2006). Proteins targeted to the thylakoids need further assistance to reach the lumen. Four major pathways have been described for proteins to insert into or transport across the thylakoid membrane: the spontaneous and signal recognition particle/Albino3 (SRP/Alb3) pathways for integrating membrane proteins, and twin arginine translocation (Tat) and the Secretory (Sec or Sec1) for transport of lumen localized proteins (Celedon and Cline 2013). A second Sec pathway (Sec2) has been postulated and although its substrates remain to be clearly demonstrated, it seems to be another protein route essential for plastid biogenesis (Skalitzky et al. 2011).

The SRP/Alb3 pathway facilitate integration of the membrane spanning light harvesting chlorophyll a/b binding proteins (Pribil et al. 2014), but how and with which translocase other multispanning proteins such as TatC and SecY1 are integrated is currently not known (Celedon and Cline 2013). Although TatC has been shown to use the Sec system in *E. coli*, no support exists for TatC using any of the four best known thylakoid targeting pathways (Celedon and Cline 2013). Interestingly, the Sec2 pathway includes SecY2 and reduced expression of SecY2 using RNAi mutants showed reduced level of e.g., TatC and SecY1, which implies that these proteins may be substrates of the Sec2 pathway (Skalitzky et al. 2011).

A model has been proposed where the TOC and TIC translocases at the envelope cooperate with the Sec2 pathway at the inner envelope. This co-operative mechanism would secure integration of e.g., TatC and SecY1 and other multispanning proteins at the inner envelope membrane for further transport to the thylakoid membrane. Thus, either invaginations or vesicles would be needed for the proteins to move to their final destination (Celedon and Cline 2013). However, if the model is correct, a sorting mechanism at the inner envelope must exist to select for further transport of multispanning thylakoid proteins. Vesicles may serve this role, as bioinformatics predict cargo selecting proteins to be present within chloroplasts (Khan et al. 2013). Thus, vesicles are suggested to operate in an additional thylakoid targeting pathway (Garcia et al. 2010; Khan et al. 2013; Vothknecht and Westhoff 2001).

Light harvesting complex proteins were identified as putative cargos of chloroplast vesicles (Khan et al. 2013), and as interactors to CPRabA5e using a yeast two-hybrid assay (Karim et al. 2014) Moreover, SCO2 interacts with a light harvesting complex protein both in vitro and in vivo but not with SRP54 or FtsY of the SRP/Alb3 pathway. Hence, it was proposed that the SRP/Alb3 pathway was mainly for transport of the light harvesting complex protein at the rosette stage whereas vesicle transport occurred preferably at the cotyledon stage (Tanz et al. 2012). Moreover, homozygous single and double mutants of the SRP/Alb3 pathway are still viable, which raises the question of an alternative pathway (Tanz et al. 2012). Accordingly, while light harvesting complex proteins have traditionally been considered as SRP/Alb3 travelers it is now challenged by vesicle transport. Interestingly, light harvesting complex proteins in the single cell green alga *Chlamydomonas reinhardtii* have been suggested to use vesicle transport, as the proliferation of vesicles coincides with transport of these proteins (Eggink et al. 2001; Hoober 1972; Hoober et al. 1991; Lindquist and Aronsson 2014; Tanz et al. 2012).

VIPP has been observed to co-sediment with a TOC component and actin (Jouhet and Gray 2009a). This could be another plausible route involving import and vesicle transport for thylakoid targeted proteins (Jouhet and Gray 2009b). That VIPP1 can form microtubule-like structures would suggest that VIPP1 could act as a vesicle track, which is consistent with the aberrant thylakoids found in mutants lacking VIPP1 (Liu et al. 2007). While a cytoskeletal structure is not confirmed in chloroplasts there are reports of microtubule-like structures not only in chloroplast but also other plastid types (Solymosi and Keresztes 2012). It has been suggested that vesicles in general require microtubules for long distances whereas short distances make use of actin or possibly even diffusion (Kamal and Goldstein 2000). However, in chloroplasts the distance between the envelope and the thylakoid membrane is rather short, being less than 300 nm (Lindquist et al. 2016). Thus, the lack of a chloroplast cytoskeleton may be of less concern for vesicle transport.

## Conclusion

Vesicles are present in chloroplasts, as well as in other plastids (Lindquist et al. 2016), and even though the system has been predicted to be similar to the cytosolic, and especially the COPII system, several components are missing. In fact, only two of the predicted putative COPII homologs have been characterized so far in chloroplasts, whereas the other proteins suggested to be involved in vesicle transport in chloroplasts are not even related to the COPII or the COPI/CCV system. Thus, despite similarities with COPII and that vesicles can be e.g., inhibited with same inhibitors as for the cytosolic system, the chloroplast vesicle system cannot be said to be exclusively of eukaryotic origin (Westphal et al. 2001b, 2003); e.g., CPSAR1 and VIPP are suggested to have a prokaryotic origin. Thus, the chloroplast vesicle system is built up by both eukaryotic and prokaryotic components, being most likely a unique system. If one assumes that the chloroplast vesicle system was adopted from the cytosol of its endosymbiotic host, it could have needed modifications which prokaryotic proteins may have evolved to fulfill. If so, it would be similar to the protein import machinery where TOC and TIC protein components are of both prokaryotic and eukaryotic origin (Celedon and Cline 2013; Keeling 2013; Vothknecht and Soll 2007). The evolutionary benefit of a chloroplast vesicle system could be to gain a repair system, and/or to provide capacity to remodel thylakoid membranes in response to environmental cues, an important consideration given the extended life span of a plant compared to that of cyanobacteria.
